# Production and Evaluation of *Toxoplasma gondii* Recombinant Surface Antigen 1 (SAG1) for Serodiagnosis of Acute and Chronic *Toxoplasma* Infection in Human Sera

**Published:** 2012

**Authors:** M (Mina) Selseleh, H Keshavarz, M Mohebali, S Shojaee, MH Modarressi, MR Eshragian, M (Monavar) Selseleh

**Affiliations:** 1 Dept. of Medical Parasitology and Mycology, School of Public Health, Tehran University of Medical Sciences, Tehran, Iran; 2 Center for Research of Endemic Parasites of Iran (CREPI), Tehran University of Medical Sciences, Tehran, Iran; 3 Genetic Faculty, School of Public Health, Tehran University of Medical Sciences, Tehran, Iran; 4 Epidemiology and Biostatistics Department, School of Public Health, Tehran University of Medical Sciences, Tehran, Iran

**Keywords:** *Toxoplasma gondii*, Recombinant SAG1, Acute toxoplasmosis, Chronic infection

## Abstract

**Background:**

The assays currently available for the detection of specific anti-*Toxoplasma* antibodies may vary in their abilities to detect serum immunoglobulins, due to the Lack of a purified standardized antigen. The aim of this study was evaluation the recombinant *Toxoplasma gondii* SAG1 antigen for the serodiagnosis of acute and chronic toxoplasmosis.

**Methods:**

This study describes an ELISA using recombinant SAG1 for detection of IgM and IgG antibodies against *Toxoplasma gondii* in human sera. Genomic DNA of *T. gondii* (RH Strain) was isolated and PCR reaction was performed. Recovered DNA was cloned into PTZ57R cloning vector. The recombinant plasmid was detected by restriction analysis. The SAG1 gene was subcloned in the pET- 28a expression vector. Protein production was then induced with 1 mM isopropyl-D – thiogalactopyranoside (IPTG). A total of 204 sera were tested using a commercial IgG and IgM ELISA kit (Trinity, USA) as gold standard prior to testing them with the recombinant antigen.

**Results:**

Tested sera were divided into the following groups:(a) The 74 *T. gondii* IgG positive (b) 70 *T.gondii* IgM positive (c) 60 sera who had no serological evidence of toxoplasmosis as negative sera.To determine the specificity of the test, we used other parasitic diseases including echinococusis (N=5), malaria (N=14), leishmaniasis (N=7),fasciolasis (N=4), sterengyloidiasis (N=1). Sensitivity and specificity of the generated recombinant IgG ELISA in comparison with commercial ELISA (Com ELISA) were 93% and 95%, and the sensitivity and specificity of the generated recombinant IgM ELISA were 87% and 95% respectively.

**Conclusion:**

The results acquired here show that this antigen is useful for diagnostic purposes and could be replaced by lysed, whole cell antigens for diagnosis of chronic toxoplasmosis.

## Introduction

Most infections with *Toxoplasma gondii* in humans are asymptomatic although primary infection acquired during gestation can be transmitted to the fetus through the placenta and may cause miscarriage, permanent neurological damage, premature birth and visual impairment(1). In patients such as those with acquired immunodeficiency syndrome, toxoplasmic encephalitis can be life threaten (1).

The common tests for toxoplasmosis diagnosis are mostly serological assays. Although they give satisfying results, accurate differentiation between recently acquired and chronic toxoplasmosis is very difficult. False positive reactions with antinuclear antibodies, rheumatoid factors, or naturally occurring human antibodies and false negative reactivity due to competitive inhibition by high levels of specific IgG antibodies have been described (2).

The assays currently available for the detection of specific anti *Toxoplasma* antibodies may vary in their abilities to detect serum immunoglobulins, due to the lack of a purified standardized *Toxoplasma* antigen or standard methods for preparation of the antigen. Specificity and sensitivity of these methods depend mostly on diagnostic antigens and often the early recognition of the infection or precise distinction between phases of *Toxoplasma* invasion is difficult. This is due to the fact that *T. gondii* is obligatory intracellular parasite and, hence, antigens always contaminated with non parasitic materials from culture media in which the parasite is grown. The methods of producing tachyzoites as well as antigens may vary between laboratories (3).

Therefore recombinant antigens were considered to replace the antigen obtained from lysed whole parasites. The use of recombinant antigens would allow better standardization of the tests and reduce the costs of production. In spite of potential advantages of using recombinant antigens in serology tests, only a limited number of studies have used these antigens in ElISA (4) The major advantages of recombinant antigens for the diagnosis of *T. gondii* infections are (a) the antigen composition of the test is precisely known, (b) more than one defined antigen can be used and (c) the method can be easily standardized (4).

SAG1 or P30 protein has an apparent molecular weight of 30 kDa (5) and is stage specific,being detected only in the tachyzoite stage, but absent in the sporozoite and bradyzoite stages (6, 7). This antigen is abundant on the surface of both extracellular and intracellular tachyzoites (6). SAG1 is one of the most immunogenic *T. gondii* antigens (4). SAG1 is considered as an important candidate for the development of diagnostic reagents or subunit vaccines that induce an immunodominant response (6). This antigen is suitable for use in diagnostic systems for detecting anti SAG1 specific IgG and IgM antibodies. SAG1 has no cross reactivity with proteins from other microorganism (8). Gene coding SAG1 occurs as a single copy, without introns (9, 10) and is highly conserved in *T. gondii* strains (11, 6).

The aim of this study was to evaluate the usefulness of this recombinant antigen for serodiagnosis of acute and chronic toxoplasmosis in human sera.

## Materials and Methods

### Preparation of antigens

The tachyzoites of *T. gondii*, RH strain were inoculated in peritoneal cavity of BALB/c mice. After three days the parasites were collected, washed and resuspended in phosphate buffered saline (PBS, pH 7.2). Genomic DNA of *T. gondii* RH Strain was isolated by conventional phenol, chloroform, ethanol precipitation method (12).

### PCR reaction

Genomic DNA isolated from tachyzoites was used as a template to amplify the SAG1 gene by PCR reaction.A pair of primer based on SAG1 gene sequence was designed with Eco R1 and xho1 restriction sites.

SAG1F(EcoR1):5-GAATTCATGTCGGTTTCGCTGCACC-3

SAG1R (Xho1): 5- CTCGAGCGCGACACAAGCTGCGAT-3

PCR reaction was performed in a total volume of 50 µl using 50ng DNA, 1.5 µl forward and reverse primers at 10 pmol, 50 mM Mgcl_2_, 200 µM d NTP, 10x PCR buffer, 2.5 u Taq polymerase. PCR reaction was carried out with 30 cycles of denaturation at 94°C for 40 seconds, annealing at 58°C for 60 seconds and extension at 72°C for 60 seconds. Reaction was incubated at 94°C for 5 min before beginning the PCR cycle, and it ended with a final extension at 72°C for 10 min in a thermal cycler (Corbet, Berlin, Germany).

### Gene cloning

The amplified DNA of SAG1 gene was visualized on 1% agarose gel stained with ethidium bromide then DNA band was cut and recovered by DNA purification kit (Fermentas, Germany). Recovered DNA was cloned into PTZ57R cloning vector (Fermentas) via T/A PCR product cloning kit (Fermentas) according to the manufacturer's protocol (13). The ligation reaction was transformed in *E. coli* XL1-blue strain competent cells (14) and dispensed on agar plate containing 100µg/ml ampicillin. Bacterial colonies were screened by agar plate containing X-gal (Fermentas) and IPTG (Fermentas) to discriminate between recombinant (white) and non recombinant (blue) containing ones (15). The recombinant plasmid was detected by restriction analysis with BamH1 and Not1 enzymes (16) and theSAG1 fragment was extracted from 1% agarose gel by DNA purification kit (Fermentas). The SAG1 gene was subcloned in the pET- 28a expression vector. Reaction was transformed in *E. coli* Top10F with Kanamycine and colonies contained recombinant plasmids were mass cultured on LB medium. The plasmid with the correct insert was confirmed by restriction enzymes and PCR analysis.

### Production and purification of recombinant His-6 tagged antigens

*Escherichia coli* strain Top10F containing pET28a-SAG1 was grown with vigorous shaking (250 RPM) at 37°C in liquid broth (LB) with Kanamycine to an optical density at OD 0.600. Protein production was then induced with 1 mM isopropyl-D – thi-ogalactopyranoside (IPTG) and the cells incubated with shaking at 37 °C for an additional 4h. SDS-PAGE with 12% acrylamide gel was performed. *E. coli*-SAG1 without IPTG and E.coli-SAG1 with IPTG was compared and then induced band was surveyed in comparison with uninduced band. Purification procedure by Ni-NTA purification system (Invitrogen,USA) was carried out according to the manufacturer's protocol. For this, 8 ml of lysate was prepared under native conditions and added to a prepared purification column. Settled the resin by gravity and carefully aspirated the supernatant and saved for SDS-PAGE analysis, washed with 8 ml native wash buffer (pH=8) again settled the resin by gravity and carefully aspirated the supernatant and saved for SDS- PAGE analysis. The protein concentration was determined by the Biophotometer (Eppendorf, Berlin, Germany). The recombinant protein (His6-SAG1) was identified by the SDS- PAGE and stained with Coomassie or were used for western blots. For western blots, proteins were transferred on to nitrocellulose membranes (Biotech). After transfer, the nitrocellulose membrane was blocked in a blocking solution (Skimmed milk) for 1 h at room temperature. After washing, strips of nitrocellulose membrane were incubated with IgG positive *T. gondii* human sera diluted 1:50 in skimmed milk. After washing, strips were incubated for 1 h at room temperature with rabbit anti human IgG conjugate (Diluted 1:500 in skimmed milk). After adding substrate the reaction was stopped by washing in distilled water.

### Sera

Two hundred and four serum samples were collected from different laboratories in Tehran. Thirty of them had clinical symptoms for example fever and lymphadenopathy. A total of seventy IgM positive sera, 36 sera were *T. gondii* IgG and IgM positive as well as 74 IgG positive sera and 60 sera from subjects who were not infected with *T. gondii* and were IgG and IgM negative (30 health subjects, 30 the other diseases) were examined. At first the sera tested by the *Toxoplasma* IgG ELISA kit (Trinity Biotech, USA) and *Toxoplasma* IgM ELISA kit (Trinity Biotech, USA) as gold standard. Tested sera were divided into the following groups: a) The 74 *T. gondii* IgG positive: b) 70 *T. gondii* IgM positive, However, IgM antibodies to *T. gondii* may be detectable for as early as two months in some individuals and for more than one year in others (17): c) 60 sera that had no serological evidence of toxoplasmosis. To check the recombinant antigens for cross-reactivity with heterologous antibodies, we also included sera from 14 patients infected with *Malaria*, 5 infected with *Echinococcus granulosus*, 4 infected with *Fasciola hepatica*, 1 infected with *Strongyloides stercoralis*, 6 infected with *Leishmania*. All of these sera were negative for IgM and IgG *Toxoplasma* antibodies.

This study was approved by Ethical Committee of Tehran University of Medical Sciences, as well as written informed consent was obtained from the participants.

### Determination of optimal assay conditions

The optimal working dilution of recombinant antigen and of conjugate was determined by checkerboard assays using serial dilutions of antigen, sera and conjugate. For determination of the optimal serum dilution, sera were titrated from 1:10 to 1:1280. The serum dilution that showed the highest difference in optical densities (OD) measured between positive and negative sera were selected for screening of all the sera. The sera were tested duplicate by the rSAG1- IgG and rSAG1-IgM ELISA and the mean absorbance value was calculated.

### ELISA

A total of 204 sera were tested using a commercial ELISA kit (Trinity, USA) as gold standard prior to testing them with the recombinant antigen in ELISA. Using the results of com-ELISA, sera were classified as negative, IgG positive and IgM and IgG positive for *T. gondii*. Purified recombinant antigen was individually diluted to the optimized concentration of 5-7/5 µg per ml in bicarbonate buffer (pH=8) and 0.1 ml of each antigen was added to separate wells of micro titer plates. Plates were washed with PBST, blocked with blocking buffer. Serums were diluted (1:100) for IgG antibody ELISA and (1:20) for IgM antibody surveys. After adding of diluted sera, plates were incubated then washed with PBST. IgG antibodies were detected by adding anti- human IgG conjugated with horseradish peroxidase (Dako, Denmark). As well as IgM antibodies were detected by using anti human IgM horseradish peroxidase labeled conjugates diluted. After incubation the plates were washed then the chromogenic substrate orthophenylene diamine (Merck) was added. The reaction was stopped by adding 1 M sulfuric acid and the optical density was read by an ELISA reader (Lab system, Finland) at 492 nm. The cut off value was set equal to the average OD value of the negative population plus three standard deviations. The optical density more than cut off and less than cut off were considered as positive and negative respectively.

### Statistical analysis

Sensitivity and specificity obtained from TP/TP + FN *100 and TN/TN + FP *100 formula respectively (3).

## Results

**Fig. 1:**
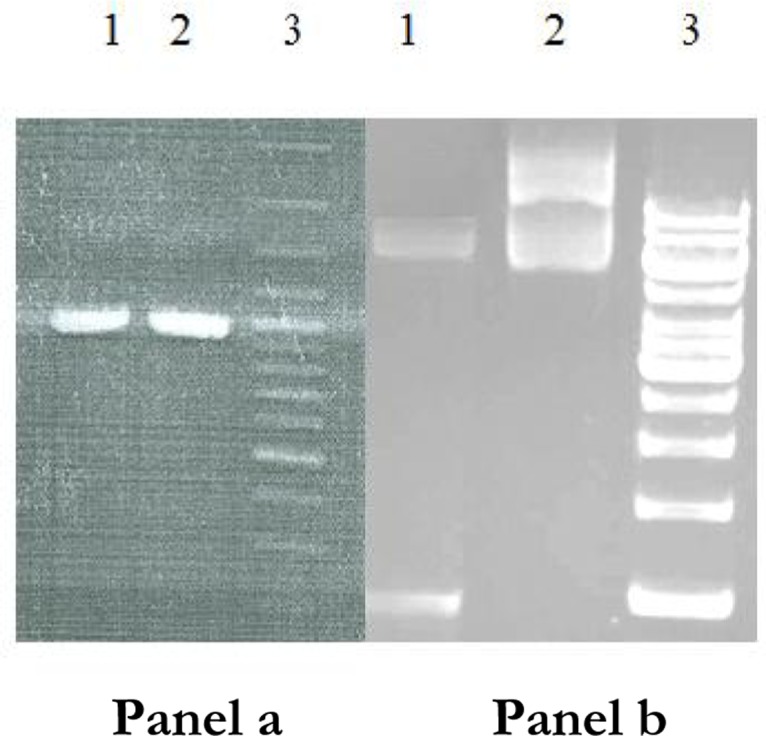
Confirmation of subcloning. A PCR was performed using Pet28a-SAG1 with SAG1 primers (1000bp) ( panel a).The reaction of confirmation enzyme is shown(panel b). Panel a: lane 1, 2 SAG1 gene (1000 bp); lane 3, DNA ladder. Panel b: lane 1 digested Pet28a-SAG1 (1000 bp)-lane 2 uncut Pet28a-lane 3 DNA ladder (1Kb)

The SAG1 gene was subcloned into pET-28a and recombinant plasmid confirmed by PCR andenzyme digestion (Fig. 1). Recombinant antigen was produced in bacteria by inducing with one mM isopropyl-D–thiogalactopyranoside (IPTG) (Fig. 2a).The protein was purified using Ni-NTA column. In SDS-PAGE analysis rSAG1 was found to resolve at 30 kDa (Fig. 2b). Around 7mg of His-tag-SAG1 was purified from 100 ml of induced culture. The western blot result is presented in (Fig. 3).

**Fig. 2a:**
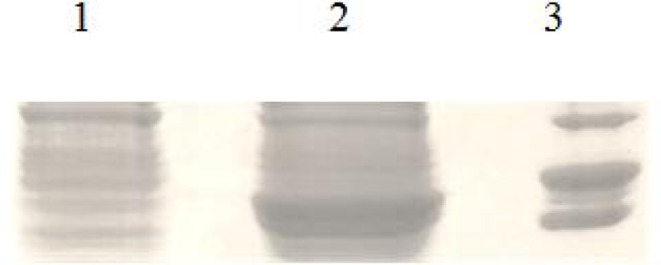
SDS-PAGE analysis of rSAG1 expression using 12% acrylamide gel. Lane 1 uninduced culture, Lane 2 expression after 7h of induction (30kDa), Lane 3 molecular protein marker

**Fig. 2b:**
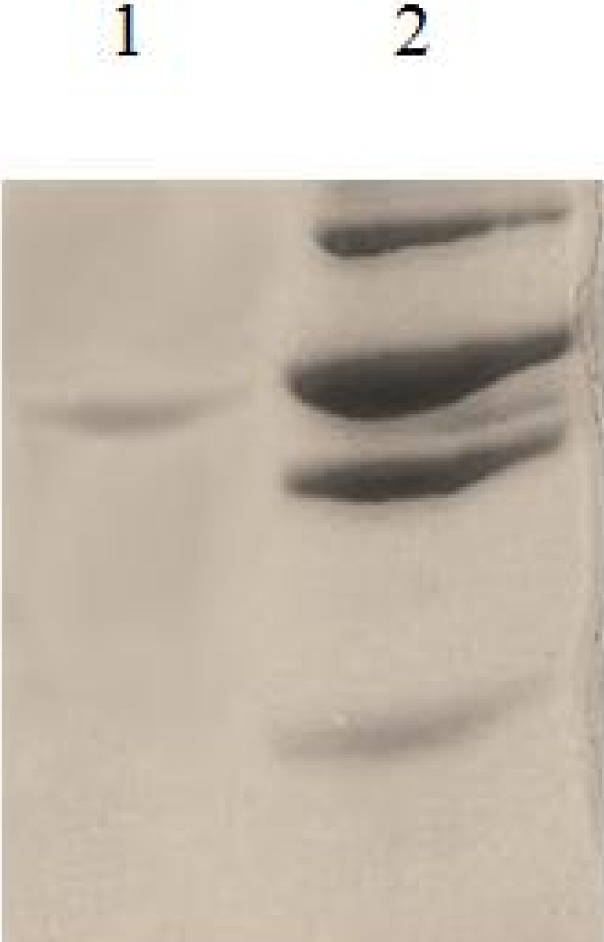
SDS-PAGE analysis of purified recombinant SAG1 protein using 12% acrylamide gel. Lane 1: purified rSAG1 protein (30kDa), lane2: molecular protein marker

**Fig. 3:**
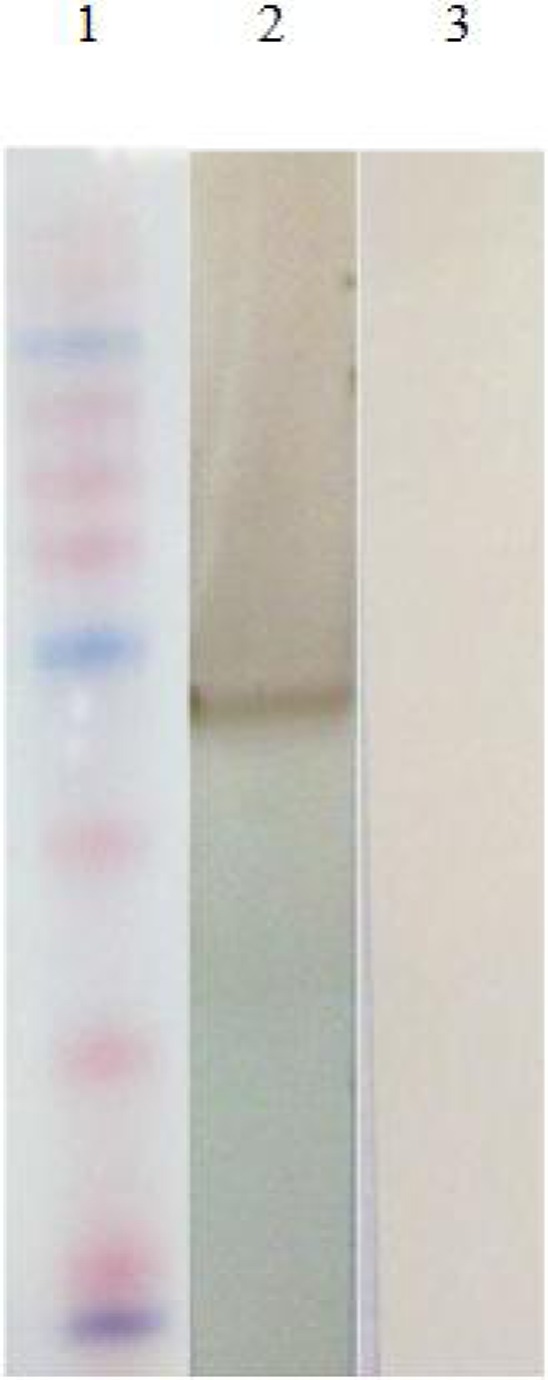
Western blot analysis of the rSAG1 protein using a rabbit anti human IgG conjugate. Lane 1, molecular protein marker, Lane 2) purified rSAG1 protein (30kD) Lane 3) induced control culture of cells lacking the SAG1 insert

The western blotting result showed a reaction against antigen of 30 kDa for SAG1. We classified sera that contained IgM antibodies against *Toxoplasma* antigens as acute toxoplasmosis. Sera that contained only IgG antibodies to *Toxoplasma* antigens were considered as chronic toxoplasmosis. The checkerboard assay with rSAG1, determined a working dilution of 7.5 µg/ml recombinant antigen per well for sera from acute toxoplasmosis and 5µg/ml for sera from chronic toxoplasmosis and 1:500 for the conjugate. OD values obtained with serial dilutions of the positive and negative sera under the optimal assay conditions. A serum dilution of 1:20 for acute toxoplasmosis and 1:100 for chronic toxoplasmosis were selected for the screening of single sera as this dilution revealed the highest difference in OD values between positive and negative sera. The sensitivity and specificity of this test using sera from patient with acute toxoplasmosis was 87% and 95% respectively. The sensitivity and specificity of this test using sera from patient with chronic toxoplasmosis was 93% and 95% respectively. The results of Com-ELISA in comparison recombinant ELISA were shown in (Table 1).

**Table 1:** The results of Com- IgG ELISA and Com-IgM ELISA in comparison recombinant ELISA

Individual Category	No of samples * Com(ELISA)	SAG1 recombinant Antigen	
		No. of positive samples	%
I-*Toxoplasma* infection		
- Acute	70	61	87
-Chronic	74	69	93
П-Non-*Toxoplasma* infection		
(Healthy individuals)	30	3	10
other infection disease	30	0	0
Total	204		

* Commercial ELISA

Mean absorbance values, standard deviations, and minimal and maximal absorbance in IgM and IgG recombinant ELISA were shown in (Table 2).

**Table 2:** Mean absorbance values, standard deviations, and minimal and maximal absorbance in IgM and IgG recombinant ELISA

Group	M (OD)	SD	Minimal absorbance value	Maximal absorbance value
Non *Toxoplasma* Infection/ n=30	0.177	0.160	0.046	0.650
Chronic *Toxoplasma* infection (IgG recombinant ELISA)/n=74	1.29	0.721	0.100	2.4
Acute Toxoplasmosis (IgM recombinant ELISA)/n=70	0.501	0.239	0.046	2.4
The other diseases n=30	0.091	0.021	0.062	2.4

## Discussion

Diagnostics methods of *T. gondii* infection sometimes are unsatisfactory. A precise distinction between acute and chronic toxoplasmosis is difficult because IgM may be present in sera for many years. This problem necessitates development of an alternative and more reliable diagnostic method using recombinant antigens (17, 18). In this paper for acute phase sera the sensitivity and specificity rSAG1 was 87% and 95% respectively. For chronic phase sensitivity and specificity was 93% and 95% respectively. In this study, the DNA sequence encoding fragment of *T. gondii* SAG1 was cloned and expressed under T7 promoter based pET-28a expression vector. This vector has a lot of advantages; many pET vectors have the advantage of carrying the His Tag sequence. The His·Tag sequence binds to divalent captions (e.g., Ni2 + ) immobilized on the His Bind metal chelation resin (19).We decided to use an expression plasmid with a short His fusion tag to the recombinant protein to prevent possible nonspecific reaction of rSAG1 and proteins of serum in ELISA experiments.

Jalallou et al. (20) expressed SAG1 in pET32a and used rSAG1 for detection *T. gondii* specific IgG in human sera by ELISA. Sensitivity and specificity were 88.4% and 88% respectively. In this survey they did not use from IgM positive sera for surveying the rSAG1, while in our study this recombinant antigen was surveyed with IgG and IgM positive sera.Velmurugan et al. (21) expressed SAG1 and GRA7 in pET-32(b) and pET-32(c) as His-tag-thirodoxin fusion proteins, in an insoluble form and transformed into BL21 *E. coli*. They used from these recombinant antigens in serodiagnosis of goat toxoplasmosis (Izatnagar isolate). In their study sensitivity and specificity for rSAG1was 83% and 88.4% respectively. These results confirmed our study that carried out on human sera.

Buffolano et al. (22) previously reported that SAG1 reacted with 75% of the sera from congenitally infected infants. In another survey (23) rSAG1 rec- ELISA could detect 83% of IgG antibodies in positive IgG sera. They used from 24 IgG positive and 19 IgM positive samples. It seems that the number of samples is not enough for deduction. Pietkiewiez et al. (24) used sera from patients with chronic toxoplasmosis and showed that increasing the level of antibody titers increased the ability of r SAG1 to detect positive sera. These results are consistent with our findings. In contrast, Nigro et al. (25) showed low or no reactivity with rSAG1. It seems they used a truncated gene and a purification method that resulted in incorrect folding recombinant protein. Nigro showed similar sensitivity values for rSAG1and rROP2for both chronic and recently infected groups of animals suggesting that these recombinant proteins are not useful as serological markers to discriminate between these two infected groups.

Difference sensitivity rates between these researches and the present study might be related to use of various vectors and quality of purification recombinant antigens or protein folding. The immunogenic nature of rSAG1 protein is argued when it is denatured as it is a conformational protein and its immunogenicity is based on the correct folding of the protein (26). In the case of disulfide-bonded proteins, inclusion body formation is more likely, where the protein is produced in the bacterial cytosol and the reducing cellular compartment is not favorable for the formation of disulfide bonds. As a result, aggregation of improperly folded protein is not deniable (27). The optimization of some growth elements, like the use of low temperature and non saturating amount of the expression inducer improve the yield of soluble recombinant proteins (21).

Our results showed that recombinant *T. gondii* rSAG1 had a high specificity for antibodies to *T. gondii*. The rSAG1 did not react with the sera from humans who were not infected with *T. gondii*, including those who suffered from other parasitic diseases. It shows that our rSAG1 does not have any similarity with other parasitic antigens; consequently it leads to elimination of false positive results in ELISA. In the present study, the rSAG1 was expressed as soluble fraction and in large quantities to earlier studies (28). The high yield obtained is due to controlled expression of the cloned gene as well as use of TOP10 *E. coli* that provided high transformation efficiency and is ideal for high-efficiency cloning and plasmid propagation.

Precise discrimination between the acute and chronic phases of toxoplasmosis in individual humans is not easy. In our results the ELISA using rSAG1 antigen showed a considerably higher sensitivity to sera from human with chronic toxoplasmosis than those from patients with acute toxoplasmosis. Therefore it seems presumably that at least some epitopes presented by SAG1 play an important role in the antibody response of the human host during chronic toxoplasmosis. In conclusion, ELISA using rSAG1described herein appears to be a useful method for the diagnosis of chronic toxoplasmosis. Identifying of SAG1 in the other strains of *Toxoplasma gondii* could be useful for developing of recombinant antigen technology in future.
